# Editorial: Infections in the intensive care unit, volume II

**DOI:** 10.3389/fmed.2025.1550303

**Published:** 2025-01-22

**Authors:** Lihui Wang, Bin Lin, Zhongheng Zhang, Yuetian Yu

**Affiliations:** ^1^Department of Critical Care Medicine, Renji Hospital, School of Medicine, Shanghai Jiao Tong University, Shanghai, China; ^2^Key Laboratory of Intelligent Pharmacy and Individualized Therapy of Huzhou, Huzhou, China; ^3^Department of Pharmacy, Changxing People's Hospital, Second Affiliated Hospital of Zhejiang University School of Medicine, Huzhou, China; ^4^Department of Emergency Medicine, Sir Run-Run Shaw Hospital, Zhejiang University School of Medicine, Hangzhou, China; ^5^Provincial Key Laboratory of Precise Diagnosis and Treatment of Abdominal Infection, Sir Run Run Shaw Hospital, Zhejiang University School of Medicine, Hangzhou, China

**Keywords:** infection, intensive care unit, sepsis, antibiotic stewardship, multidrug-resistant, multi-disciplinary treatment

Infection is one of the most serious challenges in the intensive care unit (ICU). According to data from a global multi-center cross-sectional study, almost half of the critically ill patients are admitted to ICU with infectious diseases. Furthermore, others may have ICU-acquired infection (IAI) because of invasive procedures, immunosuppression, or even inappropriate antibiotics treatment (1). Antimicrobial resistance is another problem that we have to face. It may lead to a shortage of antimicrobial agent selection and a substantial attributable mortality. Thus, applying the antimicrobial stewardship program (ASP) and infection control program (ICP) is much more essential, and the joint effort involving multi-disciplinary treatment (MDT) is needed (2).

Therefore, we have compiled this Research Topic on infection in the ICU, aiming for an in-depth discussion in the relevant fields. We are also pleased to receive over 50 submissions, of which 16 articles have been successfully published after revisions. This Research Topic mainly focuses on the following areas.

## Epidemiological trends of drug-resistant pathogens in the ICU

Global epidemiological surveys indicate that 51% of critically ill patients admitted to the ICU have concomitant infectious diseases, among which pneumonia account for 63.5%, abdominal infections for 19.6%, and bloodstream infections for 15.1%. The pathogens causing these infections are primarily non-fermenting Gram-negative bacteria, with antibiotic-resistant non-fermenters accounting for approximately 20%. Fungal infections make up 16.4% (1). The misuse of antibiotics has led to an increase in antibacterial and antifungal resistance, making the demand for new antibiotics extremely urgent.

During the COVID-19 pandemic, the abuse of antibiotics has further increased bacterial and fungal resistance. According to a special report from the CDC in 2022, in the first year of the pandemic, the detection rate of Carbapenem-resistant *Acinetobacter* increased by 78%, the detection rate of Carbapenem-resistant *Enterobacterales* rose by 35%, and Antifungal-resistant Candida increased by 26%. Most of these pathogens originated from adult ICU patients, resulting in a significant economic burden on healthcare (3). Therefore, based on factors such as resistance, therapeutic drug selectivity, and mortality rates, the World Health Organization (WHO) updated the priority lists for fungi and bacteria in 2022 and 2024, respectively, providing a basis for graded and tiered management of infectious diseases in different countries and regions.

## The optimal timing for the initiation of antimicrobial therapy and the evolution of related concepts

Any disease, if detected and treated early, will lead to better outcomes. The same is true for sepsis. For every one-hour delay in initiating antimicrobial therapy, the mortality rate of sepsis patients increases by 7.6% (4). Therefore, we recommend “Hit hard and hit early” at the outset. The guidelines for the surviving sepsis campaign (SSC) also indicate that the bundled for sepsis should be shortened from 6 h to 3 h, or even 1 h (4). However, in the current era of resistant bacteria, we should reflect on our empirical antimicrobial prescriptions. Are we sufficiently accurate in diagnosing sepsis? Do these patients truly require broad-spectrum antimicrobial treatment? If we optimize empirical antimicrobial prescriptions, can we improve the outcomes while also reducing the incidence of acute kidney injury (AKI) or clostridium difficile infections (CDI)?

In the routine diagnostic and therapeutic work in the ICU, we typically prescribe empirical antibiotics for critically patients in the following situations: (1) Host factors, clinical signs, and laboratory tests are all consistent with the guidelines for infectious diseases; (2) High-risk hosts are in a high-risk stage of infection, such as patients who have undergone allogeneic stem cell transplantation (HSCT) and develop fever 1 week after leaving the transplant unit; (3) Clinicians have rich experience but lack laboratory support, for example, 1 month after kidney transplantation, a patient develops fever, dry cough, and chest CT indicates diffuse exudation in both lungs, which may lead to the initiation of empirical treatment with trimethoprim-sulfamethoxazole for pneumocystisjirovecii pneumonia; (4) Concerns about potentially missing rapidly progressing lethal infectious diseases may prompt the initiation of antimicrobial therapy, which is something we should particularly reflect on during the COVID-19 pandemic.

Currently, the initiation of antibiotics has shifted from “when to start” to “when not to start.” Antibiotic treatment should not be initiated if the patient does not have an infectious disease. Another new concept is “watching and waiting,” which means that when the patient's vital signs are relatively stable and there is significant difficulty in differentiating sepsis, a waiting period of 3 h can be allowed for the identification of pathogens and resistance phenotypes based on rapid diagnostic test (RDT). If the pathogen detection results are still unavailable after 3 h but the patient's vital signs remain stable, the waiting time can be extended to 6 h (4). However, for patients with septic shock, due to a mortality rate close to 50%, it is still recommended to initiate broad-spectrum antibiotics within 1 h according to the bundle (5). Two points need to be emphasized during this process: (1) Assess potential pathogens and resistance based on the epidemiological situation of resistant bacteria; (2) Clearly understand the reasons for using RDT, striving for accurate interpretation of test reports.

## Selection of initial antibiotic therapy and optimize antibiotic dosing

Delayed initiation of antibiotic treatment may lead to poor prognosis in patients with severe infections. Similarly, even if antibiotic treatment is started promptly, if the pathogens causing the infection are not adequately covered, the prognosis for the patient remains poor (6). Therefore, it is necessary to infer the possible pathogens and their resistance phenotypes that could cause infection in patients based on epidemiological data. This requires not only referencing local epidemiological data on pathogen infections but also integrating the infection epidemiological data from the specific locality and department (7–9). In addition, it is necessary to further understand the patient's recent history of antibiotic use, the history of colonization by drug-resistant bacteria, whether they have undergone past decolonization treatments, and, if conditions permit, to make a comprehensive judgment based on the changes in the sensitivity breakpoints of drug-resistant bacteria (10, 11).

For immunocompromised hosts (ICHs) commonly found in the ICU, it is essential to assess their underlying diseases to determine the type of immune dysfunction and provide targeted coverage for core pathogens. If empirical treatment is ineffective, the antimicrobial spectrum should be further expanded to include common pathogens (12). Additionally, a large amount of clinical data can be generated daily in the ICU, which can be used to establish clinical prediction models. However, this should not be viewed as a “decision-making tool” but rather as an “exclusion tool” to rule out whether the patient has an infectious disease or an infection caused by resistant bacteria, thereby minimizing the unreasonable use of empirical broad-spectrum antibiotics (13).

As ICU practitioners deepen their understanding of infectious diseases and as microbial testing technologies continue to develop, the success rate of treating severely infected patients is increasing. However, there are still some patients whom we cannot cure, primarily due to the following five reasons: (1) Insufficient drainage of the infection site; (2) Presence of mixed infections where antibiotics do not fully cover the pathogens; (3) Induction of resistance during antibiotic use; (4) Excessively low host immune function levels; (5) Pharmacokinetic and pharmacodynamic (PK/PD) parameters of antibiotics failing to meet standards, which is increasingly being focused on. The pathophysiological changes in patients with severe infections in the ICU are extremely complex and dynamic. Factors such as apparent volume distribution (Vd), serum albumin levels, and the impact of extracorporeal life support devices all require continuous dynamic assessment. It is also encouraged to adjust doses precisely based on Bayesian models, leveraging big data, artificial intelligence (AI), and model-informed precision dosing (MIPD) to achieve individualized and precise treatment (14).

## De-escalation strategy and duration of antibiotics treatment

Long-term use of broad-spectrum antibiotics can affect the stability of the host's internal microecology, while also increasing the burden on the liver and kidney of patients, potentially leading to the development of antibiotic resistance. Therefore, it is necessary to encourage the early implementation of a de-escalation strategy for antibiotics, which includes using monotherapy instead of combination therapy, employing narrow-spectrum antibiotics instead of broad-spectrum ones, and discontinuing the use of antimicrobial agents that do not target the causative pathogens of infections (15). However, in the ICU, nearly 45% of cases of sepsis remain culture-negative, making the accurate implementation of de-escalation strategies quite challenging. When the culture results for suspected sepsis patients are negative, it is essential to reassess the accuracy of the sepsis diagnosis, while also relying on non-culture methods such as molecular biology testing to identify the pathogens responsible for the infection, and to timely adjust the antimicrobial treatment regimen (16, 17).

The timing for discontinuing effective antibiotic treatment regimens has long been a contentious issue. Previously, there was a strong advocacy for completing the full course to prevent the recurrence of infectious diseases. However, there is now a greater emphasis on dynamically assessing the benefits and risks of antibiotics, suggesting that antibiotics should be discontinued as early as possible once therapeutic effects are achieved, in order to minimize potential side effects. Over the past 25 years, at least 45 randomized controlled trials (RCTs) have compared the efficacy of short vs. long courses of treatment for infectious diseases, including community-acquired pneumonia, intra-abdominal infections, cellulitis (18). Most studies indicate that there is no increase in the recurrence or mortality rates of diseases when comparing short courses to long courses. Consequently, the 2021 guidelines from the SSC recommend short courses for non-immunocompromised hosts with well-drained infections who respond clinically well to treatment, including pneumonia, bacterial blood stream infection, intra-abdominal infections, and urinary tract infections. No recommendations for short courses currently exist for conditions such as tuberculosis, osteomyelitis, and invasive fungal infections (IFI).

## Summary of the Research Topic

In this Research Topic, “*Infections in the Intensive Care Unit, Volume II*,” we have assembled a collection of articles that delve into the complexities of managing infections within the ICU. The contributions in this volume encompass a wide array of topics, from the application of novel diagnostic tools to the challenges of treating multidrug-resistant bacteria. Each article provides a comprehensive analysis of the current state of knowledge, offering insights into the latest strategies for diagnosing and treating infections in critically ill patients. The articles also highlight the importance of antimicrobial stewardship and the role of precision medicine in improving patient outcomes. Looking ahead, the prospects for research in ICU infections are promising, with a focus on leveraging technology and data to enhance diagnostic accuracy and treatment efficacy. The integration of artificial intelligence and machine learning into the analysis of clinical data holds the potential to significantly improve our ability to predict patient outcomes and personalize treatment plans. Furthermore, as new antimicrobial agents are developed, research will play a crucial role in determining their safety and efficacy in real-world settings.

The future also holds the promise of a more concerted effort in antimicrobial stewardship, aiming to preserve the effectiveness of existing antibiotics and slow the emergence of resistance. This will require collaborative research efforts across disciplines, combining the expertise of clinicians, microbiologists, pharmacists, and public health officials. We hope to provide more information related to infections in the ICU in the volume III, and we also hope that the next Research Topic will receive more support from everyone.
